# Carboxyhaemoglobin Levels among Traders Exposed to Vehicular Emissions in Three Motor Parks in Ibadan, Nigeria

**DOI:** 10.1155/2018/9174868

**Published:** 2018-06-03

**Authors:** Olusola Olabisi Ogunseye, Godson R. E. E. Ana, Daniel C. Uhiara, Derek G. Shendell

**Affiliations:** ^1^Department of Environmental Health Sciences, Faculty of Public Health, University of Ibadan, Nigeria; ^2^NJ Safe Schools Program, Rutgers School of Public Health (SPH), Piscataway, NJ, USA; ^3^Department of Epidemiology, Rutgers SPH, Piscataway, NJ, USA; ^4^Department of Environmental and Occupational Health, Rutgers SPH, Piscataway, NJ, USA; ^5^Exposure Measurement and Assessment Division, Environmental and Occupational Health Sciences Institute, Rutgers, The State University of NJ, Piscataway, NJ, USA

## Abstract

Carbon monoxide (CO) remains a leading cause of work-related chemical poisoning. Vehicular emissions are the primary daily ambient source of CO in urban Nigerian motor parks, where there have been few human exposure studies. Using a cross-sectional comparative design, we assessed carboxyhaemoglobin levels (% COHb), a biomarker of CO exposure, among traders at three motor parks (AMP, IMP, and NMP) and other traders (nonmotor park workers) in Ibadan, Nigeria, using a noninvasive pulse CO-dosimeter (Rad 57). Ninety-three traders were proportionally allocated between motor parks; 93 other traders were selected based on specific study inclusion criteria. Mean ages of motor park traders and other traders were comparable, 37.8 ± 11.1 and 38.7 ± 9.6, respectively. Mean % COHb for traders (range 3–22) at AMP, IMP, and NMP was 11.2 ± 3.8, 11.6 ± 3.1, and 12.2 ± 3.3, respectively, while mean % COHb for other traders was about three times lower, 4.1 ± 1.7 (range 2–8). Overall, mean % COHb for motor park traders, 11.7 ± 3.3, was also significantly higher than for other traders (*p* < 0.05). Nevertheless, mean % COHb for both groups exceeded the current World Health Organization guideline, 2.5%. This study suggested that motor park traders have higher % COHb and thus are highly susceptible to exposure and more vulnerable to known risks of adverse health effects from exposure to CO.

## 1. Introduction

Air pollution is an important public health problem in most cities in less developed countries (LDCs) [1]. Air pollution is the release of substances in their chemical, particulate, or biological states into the atmosphere which causes harm or discomfort to human beings and other living organisms or brings about damage to the natural or built environment [2]. Examples of such pollutant gases and particles include nitrogen dioxide (NO_2_), sulphur dioxide (SO_2_), carbon monoxide (CO), particulate matter (PM), ozone, and microorganisms, along with a variety of airborne heavy metals and hydrocarbons [3]. Carbon monoxide (CO), unburned hydrocarbon (UHC), nitrogen oxides (NO*x*), sulphur oxides (SO*x*), and soot, in addition to volatile organic compounds (VOCs), are the main combustion products of vehicular engines [4, 5].

CO is a colorless, odourless, and nonirritating gas produced as a byproduct of incomplete combustion of carbonaceous materials [6–8], including petroleum products, coal, natural gas, wood, and plastics. CO can be produced at toxic levels by internal combustion engines, structural fires, industrial operations, and improperly vented heating or cooking appliances [6]. Strategies developed by industrialized countries aimed at controlling air pollution have focused on the regulation of CO in ambient air and in occupational settings [9].

Haemoglobin has greater affinity for CO than oxygen; hence, CO binds with haemoglobin to form a relatively stable complex called carboxyhaemoglobin (COHb). The presence of COHb in the blood reduces the oxygen carrying capacity of the blood and restricts the access of body tissues to oxygen, resulting in tissue hypoxia [9–12]. Because CO binds avidly to haemoglobin, COHb remains in the circulation for hours and it is a biomarker of recent exposure to CO [13]. In a recent occupational study in southwestern Nigeria, elevated levels of CO and PM contributed to lower lung function measures for forced expiratory volume and peak expiratory flow rate and high levels of COHb [14].

People are exposed to CO via several polluted environments such as travelling in motor vehicles, at their workplaces, visits to urban locations associated with combustion sources, and cooking and heating with domestic gas, charcoal, or wood fires and wherever there is tobacco smoke. However, the most prominent outdoor sources of CO exposure for many individuals are vehicular emissions [13]. Usually, over 90 percent of CO in city centres comes from vehicles [15]. Other anthropogenic CO sources include industrial plant exhaust, burning of waste, defective heaters, stoves, and ovens [16]. Several studies have reported CO pollution is a serious environmental issue in urban areas worldwide, especially in big cities where traffic intensity is routinely high. Certain factors influence CO levels over space and time in urban regions including traffic density, traffic congestion, the types of vehicles on the road, and meteorological conditions [17, 18]. In LDCs, outdoor, in-vehicle, and personal exposure levels of CO are generally higher than in industrialized countries due to factors such as poor vehicle maintenance and insufficient use of vehicle emission control systems [17].

CO is toxic [11, 19] and is the major cause of death from poisoning [20]. CO is responsible for more than one-half of the accidental poisonings and deaths reported throughout the world each year, and CO poisoning is the most common type of accidental poisoning in the U.S. [8, 21, 22].

Dose and time duration of exposure are factors that determine the effects and severity of health risks in CO exposure [23] and range from mild cardiovascular and neurobehavioral effects at low concentrations to unconsciousness and death after prolonged exposures or after acute exposures to high concentrations of CO. Relatively low levels of CO found in outdoor environments and polluted workplaces also present certain risks, which have been reported in several studies [9, 10, 24, 25].

Acute CO poisoning occurs as a result of the inability of body tissues to access oxygen. Signs and symptoms of CO toxicity, in order of increasing severity include headache, nausea, dilation of cutaneous vasculature, vomiting, dizziness, and blurred vision, confusion, syncope, chest pain, dyspnea, weakness, tachycardia, tachypnea rhabdomyolysis, palpitations, cardiac dysrhythmias, hypotension, myocardial ischemia, cardiac arrest, respiratory arrest, pulmonary edema, seizures, and coma [26, 27]. The degree of CO poisoning is related to the percentage of haemoglobin converted to COHb [6]. In general, signs and symptoms of acute CO poisoning can present at COHb levels ranging from 3 to 24% [26], while healthy individuals with mild CO poisoning will require medical attention when COHb levels exceed 20% [27]. CO exposures resulting in COHb levels greater than 50% are frequently fatal in a brief time [27, 28]. However, fatalities due to CO poisoning have been reported for COHb levels between 3 and 70% [27]. One recent study concerning chronic total exposure to CO via COHb levels defined CO poisoning based on medical history and COHb% of >5% [29].

Exposures to CO assessed in controlled outdoor and occupational environments represent only a fraction of total human exposure to CO. Elevated levels of CO have been recorded, for example, in motor parks and tunnels because of the accumulation of exhaust fumes [9].

There are limited data on traffic-related exposure assessment by near or on-road measurements despite a recent increase in research; challenges in exposure assessment for recent studies include differences in measuring methods and a lack of strict quality control, hence, making it difficult to compare findings between studies [17, 30].

Motor parks are common public spaces found in every urban area in Nigeria and these motor parks vary in their design, nature, environment, and services. The importance of motor parks is apparent because of the utilization of public transport systems [31]. Various activities have occurred in Nigerian motor parks which release CO and other pollutant gases with vehicular emissions [18]. Some of these activities pose health risks to motor park users and traders. Traders in motor parks spend about 6–8 hours daily in those motor parks; hence, they are at risk of possible exposure to CO arising from vehicles and other emission sources in motor parks.

This study was aimed at generating baseline information on exposure levels of traders to CO using COHb as the biomarker given limited data on exposure to CO in public transit waiting areas such as motor parks and bus stops. In Nigeria, studies on human exposure to vehicular emissions like CO have been limited. Furthermore, there are limited studies on COHb in Nigeria, and in the few available, study participants were not traders in motor parks. Previous studies on COHb were performed on smokers, commercial bus drivers, and artisans and had employed an invasive method of obtaining blood samples through venipuncture [32–34]. In contrast, the present research also draws on the fact that the pulse CO-dosimeter (Rad 57), a handheld device from Masimo Corporations, USA, with Masimo Rainbow® SET® Technology, was used to determine the blood CO levels noninvasively among study participants.

## 2. Materials and Methods

### 2.1. Study Design

This study was a cross-sectional comparative study which involved the assessment of carboxyhaemoglobin (% COHb) levels among traders in three motor parks and nonmotor park traders in Ibadan, Nigeria.

#### 2.1.1. Study Area

The study area was Ibadan, the capital of Oyo state, one of the 36 states in Nigeria. Ibadan is positioned on longitude 3°53′ east of Greenwich Meridian and latitude 7°23′ north of the equator. This ancient city is located close to forest and grassland boundary of south western Nigeria and about 145 km North East. Due to its location, Ibadan serves as a meeting point for people and products from forest and grassland areas [18].

This research was carried out in three major motor parks in Ibadan: Akinyele Motor Park (AMP) in Akinyele Local Government Area (LGA); Iwo Road Motor Park (IMP) in Ibadan North East LGA; and “New Garage” Motor Park (NMP) located in Ibadan South West LGA, within Ibadan municipal area. AMP caters for transport linking cities in northern region which includes Kaduna, Abuja, Kano, and other major cities. IMP caters for transport needs of passengers going towards eastern region comprising Benin, Warri, Port Harcourt, Uyo, Calabar, and others. NMP links other major cities in the south west which includes Lagos, Abeokuta, Epe, Ijebu-Ode, and other coastal towns. These study sites were chosen purposively as they are the major exit points to reach northern, eastern, and other western parts of the country. Many transportation devices such as taxis, mini-buses, and buses originate and terminate at these points [35].

#### 2.1.2. Study Population

The participants for this study were traders in the three motor parks and nonmotor park traders in Ibadan. Sociodemographic characteristics such as age, gender, educational status, marital status, religion, and state of origin were collected from the traders.

#### 2.1.3. Sample Size Estimation

The formula this study used [36] was

(1) $$\begin{array}{c} n_{0}=\frac{2\times {\left(Z_{\alpha }+Z_{\beta }\right)}^{2}\times P\times \left(1-P\right)}{{\left(P_{0}-P_{1}\right)}^{2}}, \end{array}$$

where

(2) $$\begin{array}{c} P=\frac{P_{0}-P_{1}}{2}. \end{array}$$

*P*_0_ is the proportion of participants in the unexposed group exhibiting the outcome of interest.

*P*_1_ is the proportion of participants in the exposed group exhibiting the outcome of interest.

*P*_0_ = 1% and *P*_1_ = 7.1% [37].

Hence, *P* = 4.05%.

Therefore

(3) n0=2×1.96+1.282×0.0405×1−0.04050.01−0.0712=219  respondents  per  group.

#### 2.1.4. Sample Size Reduction

Sample size reduction was applied because the sample size (219) was more than the population of traders in the three motor parks that satisfied the inclusion criteria for the study. As at the period of data collection, the number of traders that satisfied the inclusion criteria at AMP, Ojoo, IMP, Iwo road and NMP, Apata were 30, 77, and 54, respectively, adding up to 161. The sample size (*n*_0_) was reduced using the equation by Israel [38] below:

(4) n=n01+n0−1/Nn=2191+219−1/161=93  respondents  per  group

The final sample size (*n*) was therefore 93 respondents per group.

#### 2.1.5. Proportional Allocation and Sampling Procedure

Purposive sampling technique was used to select the three motor parks as these motor parks serve as the major exit points to reach northern, eastern, and other western parts of the country. Taxis, mini-buses, and buses originate and terminate at these points. Permission was sought and obtained from the Chairmen, National Union of Road Transport Workers (NURTW) of these parks to conduct COHb assessment of traders within the motor parks. 93 motor park traders were proportionally allocated between the three motor parks as shown in Table 1 and were selected systematically while 93 nonmotor park traders were selected based on the inclusion criteria for nonmotor park traders.

### 2.2. Data Collection

**Figure 1:**
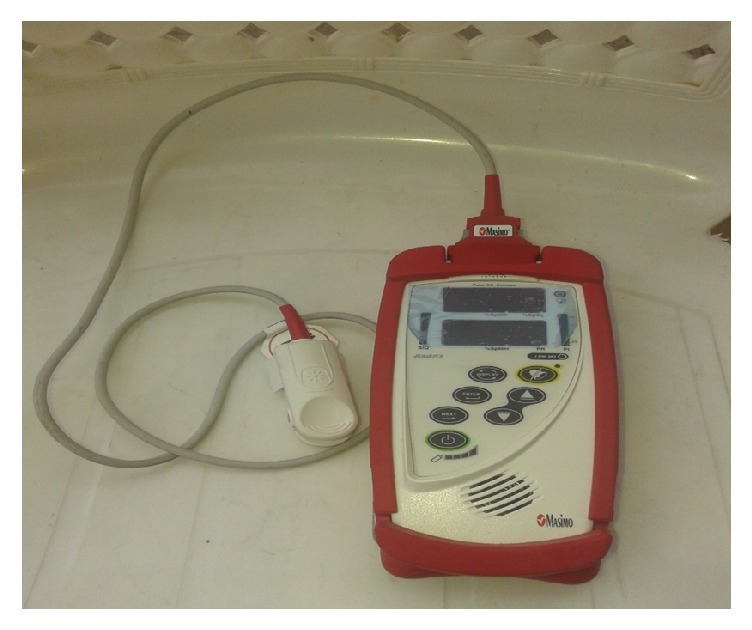
Example of noninvasive pulse CO-dosimeter (Rad 57).

**Figure 2:**
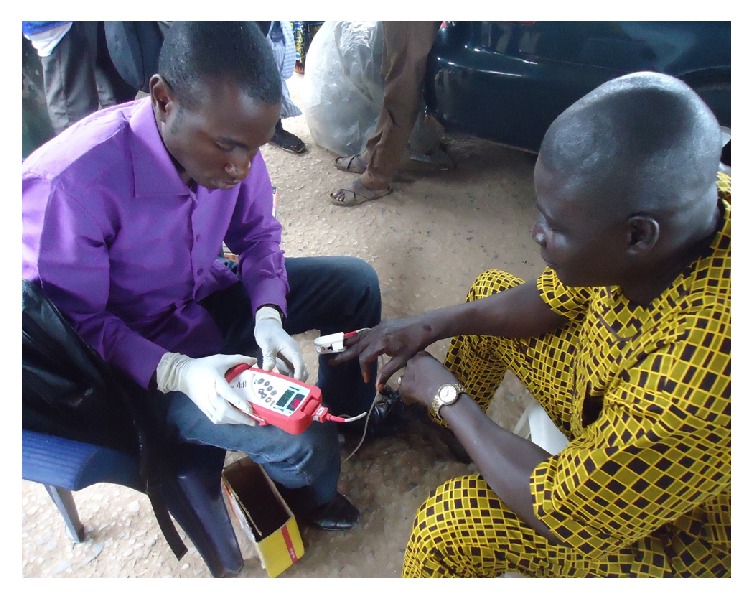
Carboxyhaemoglobin assessment of a motor park trader (photo by Olasunkanmi Williams, September, 2014).

Noninvasive pulse CO-dosimeter (Rad-57) by Masimo Corporations, USA (Figure 1), was used to assess the % COHb of study participants. The equipment comes with a sensor; each measurement was taken by placing the sensor on the fingertip of participants. Rad 57 has in-built software that automatically calibrates the equipment. Rad 57 was used to assess % COHb of the two groups of traders.  Figure 2 depicts an example of a typical COHb assessment of a trader at one study site, IMP, Iwo road.

### 2.3. Statistical Analysis

Data was entered and analysed using statistical package for the social sciences (SPSS) version 20. Descriptive and inferential statistics were used in this study. Descriptive statistics was used to summarize data. Mean ± Standard Deviation (SD) and range were calculated for % COHb of the two groups of traders (motor park traders and nonmotor park traders) and compared with WHO guideline of 2.5% [13]. Proportion of motor park traders and nonmotor park traders with % COHb higher than 2.5% was also calculated. *T*-test was used to compare % COHb levels of the two groups at 5% level of significance.

## 3. Results

### 3.1. Sociodemographic Characteristics of Motor Park Traders

Table 2 shows the sociodemographic characteristics of traders at the three sites and nonmotor park traders. For traders at AMP, Ojoo, majority of the selected traders were between 41 and 50 years (52.9%), males (100%) with secondary education (58.8%), married (64.7%), Muslims (82.4%), and natives of Oyo state (64.7%). The mean age of traders at AMP, Ojoo, was 38.3 ± 9.5. For traders at IMP, Iwo road, majority of the selected traders were between 31 and 40 years (31.1%), males (77.8%) with secondary education (42.2%), married (82.2%), Muslims (53.3%), and natives of Oyo state (57.8%). The mean age of traders at IMP, Iwo road, was 39.0 ± 10.5. For traders at NMP, Apata, majority of the selected traders were between 31 and 40 years (35.5%), males (77.8%) with secondary (38.7%) and tertiary education (38.7%), married (67.7%), Christians (48.4%), and natives of Oyo state (51.6%). The mean age of traders at NMP, Apata, was 36.1 ± 13.2. Among nonmotor park traders, majority of the selected traders were between 31 and 40 years (35.5%), female (75.3%) with secondary education (47.3%), married (88.2%), Christians (59.1%), and natives of Oyo state (54.8%). The mean age of nonmotor park traders was 38.7 ± 9.6.

### 3.2. Carboxyhaemoglobin Levels (% COHb) among Traders

**Figure 3:**
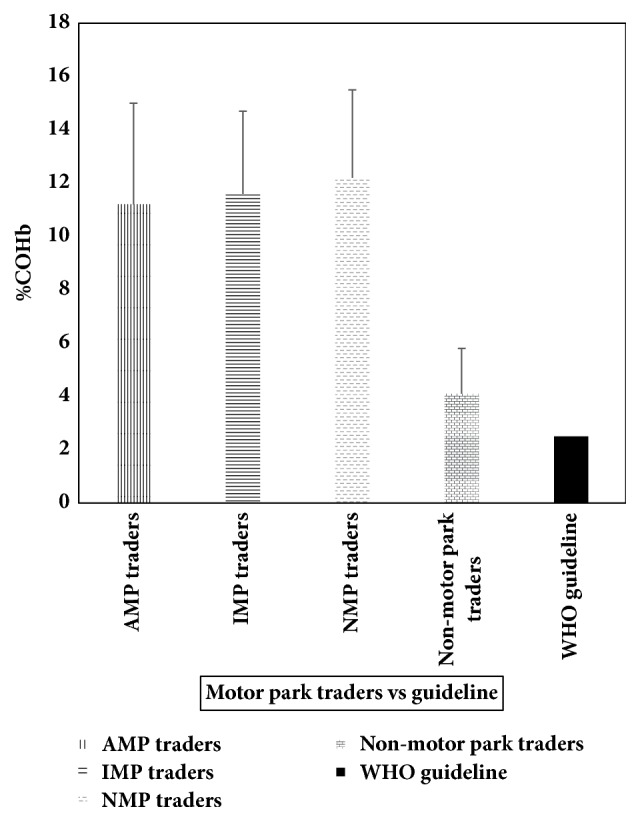
Carboxyhaemoglobin levels among study participants.

Table 3 shows the range and mean % COHb among traders at AMP, Ojoo, IMP, Iwo road, NMP, Apata, and nonmotor park traders. The ranges of % COHb were 6–19, 5–22, 3–18, and 2–8, respectively. Mean % COHb was highest among traders at NGP, Apata (12.2 ± 3.3), and lowest among traders at AMP, Ojoo (11.2 ± 3.8). The mean % COHb among motor park traders and nonmotor park traders was higher than the WHO guideline of 2.5% as shown in Figure 3. The percentage of motor park traders and nonmotor park traders with % COHb higher than 2.5% was calculated. The result showed that all (100%) of motor park traders and 76.3% of nonmotor park traders recorded % COHb higher than 2.5%.

### 3.3. Comparison of Carboxyhaemoglobin Levels between the Two Groups of Traders

Table 4 shows the comparison between mean % COHb levels among traders in the three motor parks and the comparison between aggregate mean % COHb among motor park traders and nonmotor park traders. The result showed that there was a statistically significant difference between % COHb among motor park traders and nonmotor park traders (*p* < 0.001).

We also conducted a comparison between mean % COHb among motor park traders (three sites combined) with the WHO guideline and the comparison between mean % COHb among nonmotor park traders with the WHO guideline (2.5%). There were statistically significant differences between the WHO guideline and the mean % COHb of both motor park traders and nonmotor park traders.

## 4. Discussion

The range of % COHb among traders at AMP, Ojoo, IMP, Iwo road, NMP, Apata, and nonmotor park traders was 6–19, 5–22, 3–18, and 2–8, respectively. Mean % COHb was highest among traders at NMP, Apata (12.2 ± 3.3), and lowest among traders at AMP, Ojoo (11.2 ± 3.8). The mean % COHb among motor park traders and nonmotor park traders was higher than the WHO guideline of 2.5%. The mean % COHb among motor park traders was about three times higher than that of nonmotor park traders and about four times higher than WHO guideline of 2.5%. The result also documented every motor park trader (100%) and 76.3% of nonmotor park traders recorded % COHb higher than 2.5%.

There was a statistically significant difference between % COHb among motor park and nonmotor park traders (*p* < 0.001). There was also a statistically significant difference between the WHO guideline of 2.5% and the mean % COHb of motor park traders (*p* < 0.001) and nonmotor park traders (*p* < 0.001). This result implied motor park traders have high blood CO levels and are likely to experience symptoms of CO exposure. Signs and symptoms of CO toxicity, in order of increasing severity, include (1) headache, nausea, dilation of cutaneous vasculature, vomiting, dizziness, and blurred vision; (2) confusion, syncope, chest pain, dyspnea, weakness, tachycardia, and tachypnea rhabdomyolysis; and (3) palpitations, cardiac dysrhythmias, hypotension, myocardial ischemia, cardiac arrest, respiratory arrest, pulmonary edema, seizures, and coma. In general, signs and symptoms of acute CO poisoning can present at COHb levels ranging from 3 to 24% [26].

There are several complications that may arise as a result of exposure to high CO concentration. Metabolic energy production may be impaired due to a reduction in oxygen delivery because of the elevated COHb level, exacerbated by impaired perfusion resulting from hypoxic cardiac dysfunction, which potentially impairs cellular oxidative metabolism. This occurs because hypoxia and reduction in blood flow may allow CO to bind to cytochrome c-oxidase, which inhibits aerobic adenosine triphosphate synthesis [39]. Generally, energy production and mitochondrial function are impaired in the event of high COHb. This disruption in mitochondrial electron transport also causes oxidative stress, measured as an increase in the hydroxyl-like radical fraction, and leads to the generation of hydroxyl-like radicals [40–42]. With a recorded maximum COHb level of 22% in the present study, the possibility of CO poisoning may not be ruled out.

Nonmotor park traders are also not exempted from CO exposure as their mean COHb level (4.1 ± 1.7) was higher than the WHO guideline of 2.5% and the difference was statistically significant (*p* < 0.001). Nonmotor park traders are also likely to experience mild effects of CO exposure. The elevated COHb level among nonmotor park traders may be because of CO exposure in their homes, vehicular emissions, and other combustion sources.

Other studies conducted in Nigeria were consistent with this research. In a pilot study which assessed COHb levels in some Lagos dwellers by Uko et al. [34], it was reported that these Lagos dwellers had elevated COHb concentrations ranging between 7.6 and 9.9%, with an average of 8.6%, which is severalfold higher than the WHO guideline. Furthermore, in a study which assessed COHb levels of cabinet makers (subjects) and noncabinet makers (controls) by Banjoko et al. [32], it was reported that COHb levels among cabinet makers with mean working hours of 9.5 ± 2.9 per day were 3.95% ± 1.35% while those of controls with mean working hours of 8.0 ± 0.8 per day were 2.08% ± 0.91% (*p* < 0.001). In another study which investigated ambient CO and COHb levels in Ibadan, Nigeria, by Banjoko et al. [33], COHb levels were between 0.7% and 6.5% with a mean value and standard deviation of 2.0 ± 0.68%. The results of these studies together suggested certain occupations and trades of people engaged in increased exposure to CO and consequently increased their % COHb. This is further supported in a report by WHO [13], which stated that occupational exposures up to eight hours per day, five days a week, can produce % COHb of up to about 10%.

It should be noted that the present study had some limitations. The present study focused on quantitative field measures of % COHb; we did not conduct outdoor area measures of CO, a well-known byproduct of combustion of fossil fuels, or a questionnaire about reported symptoms. An exposure biomarker like % COHb is a relatively more rigorous measure than area or even personal air concentration and self-reported survey data. In general, self-reported outcomes would be nonvalidated, subject to recall bias and possible misclassification error. Furthermore, some reported symptoms of CO exposure have other known causal agents, e.g., other chemical pollutants, infectious diseases (flu virus, illnesses like colds, etc.), and dust/particulate matter. Future studies could incorporate quantitative and qualitative data.

## 5. Conclusions

This study assessed carboxyhaemoglobin levels (% COHb) of traders in three motor parks and of nonmotor park traders in Ibadan, Nigeria. Mean % COHb among motor park traders was about three times higher than mean % COHb of nonmotor park traders and about four times higher than the current World Health Organization (WHO) guideline of 2.5%. Motor park traders had high blood carbon monoxide (CO) levels and are thus more likely to experience health risks from CO exposure and its known symptoms like headache, nausea, vomiting, dizziness, and so forth. Furthermore, given mean % COHb of both groups were above the current WHO guideline of 2.5%, nonmotor park traders are also likely to experience mild health effects of CO exposure. Therefore, this study suggests enhancing general worker awareness on adverse health effects of CO and encouraging the conduct of regular % COHb assessment, especially among motor park traders, to ascertain CO exposure levels. Reductions in vehicular emissions and improvements in monitoring and enforcement of related regulations are also warranted. Finally, controlling emissions from other known incomplete combustion-related sources contributing to outdoor air pollution at motor parks and worker homes and communities, e.g., smoking product-related activities (tobacco, e-cigarettes) and portable generators for electrical power, is recommended.

## Figures and Tables

**Table 1:** Population of traders and proportional allocation.

Motor park	Number of traders	Proportional allocation in study sample
AMP, Ojoo	30	17
IMP, Iwo road	77	45
NMP, Apata	54	31

*Total*	*161*	*93*

**Table 2:** Sociodemographic characteristics of study respondents.

Sociodemographic characteristics	Subgroups	AMP, Ojoo traders	IMP, Iwo road traders	NMP, Apata traders	Nonmotor park traders
Age	18–20	2 (11.8%)	1 (2.2%)	3 (9.7%)	3 (3.2%)
	21–30	1 (5.9%)	10 (22.2%)	7 (22.6%)	16 (17.2%)
	31–40	5 (29.4%)	14 (31.1%)	11 (35.5%)	33 (35.5%)
	41–50	9 (52.9%)	12 (26.7%)	6 (19.4%)	30 (32.3%)
	51–60	-	8 (17.8%)	2 (6.5%)	11 (11.8%)
	61–70	-	-	2 (6.5%)	-

Gender	Male	17 (100%)	35 (77.8%)	21 (67.7%)	23 (24.7%)
	Female	-	10 (22.2%)	10 (32.3%)	70 (75.3%)

Educational status	No education	1 (5.9%)	2 (4.4%)	1 (3.2%)	6 (6.5%)
	Primary education	3 (17.6%)	14 (31.1%)	6 (19.4%)	35 (37.6%)
	Secondary education	10 (58.8%)	19 (42.2%)	12 (38.7%)	44 (47.3%)
	Tertiary education	3 (17.6%)	10 (22.2%)	12 (38.7%)	8 (8.6%)

Marital status	Single	6 (35.3%)	7 (15.6%)	12 (38.7%)	11 (11.8%)
	Married	11 (64.7%)	37 (82.2%)	21 (67.7%)	82 (88.2%)
	Widow/widower	-	1 (2.2%)	-	-

Religion	Christianity	3 (17.6%)	21 (46.7%)	15 (48.4%)	55 (59.1%)
	Islam	14 (82.4%)	24 (53.3%)	13 (41.9%)	38 (40.9%)
	Traditional	-	-	2 (6.4%)	-
	No religion	-	-	1 (3.2%)	-

State of origin	Abia	-	-	1 (3.2%)	1 (1.1%)
	Benue	-	1 (2.2%)	-	-
	Cross Rivers	-	1 (2.2%)	-	-
	Delta	-	1 (2.2%)	-	1 (1.1%)
	Edo	1 (5.9%)	-	-	3 (3.2%)
	Ekiti	-	-	2 (6.5%)	4 (4.3%)
	Kogi	-	-	-	1 (1.1%)
	Kwara	1 (5.9%)	1 (2.2%)	1 (3.2%)	-
	Lagos	-	1 (2.2%)	-	-
	Ogun	-	3 (6.7%)	8 (25.8%)	15 (16.1%)
	Ondo	-	2 (4.4%)	-	7 (7.5%)
	Osun	4 (23.5%)	9 (20.0%)	3 (9.7%)	10 (10.8%)
	Oyo	11 (64.7%)	26 (57.8%)	16 (51.6%)	51 (54.8%)

**Table 3:** Mean and range of % COHb among study participants.

AMP traders	IMP traders	NMP traders	Nonmotor park traders
11.2 ± 3.8	11.6 ± 3.1	12.2 ± 3.3	4.1 ± 1.7
6–19	5–22	3–18	2–8

**Table 4:** Comparison of % COHb among study participants within and between groups.

	AMP traders	IMP traders	NMP traders	Nonmotor park traders	*f*-value	*p* value

% COHb	11.2 ± 3.8	11.6 ± 3.1	12.2 ± 3.3		0.573	0.566
% COHb (group mean)		11.7 ± 3.3		4.1 ± 1.7	24.153	<**0.001**
